# Tensor Facia Lata-iliac crest osteocutaneous flap for orbitomaxillary reconstruction: A preliminary report

**DOI:** 10.4103/0970-0358.63940

**Published:** 2010

**Authors:** Subramania Iyer, Moni A. Kuriakose

**Affiliations:** Department of Plastic and Reconstructive Surgery and Head and Neck Institute, Amrita Institute of Medical Sciences, Kochi, Kerala, India

**Keywords:** Free bone flaps, head and neck reconstruction, TFL-iliac crest flap

## Abstract

Tensor Fascia Lata muscle and musculocutaneous flap has been used in the past for reconstruction of trunk defects and also as a free flap for soft tissue reconstruction elsewhere in the body. Transferring the iliac crest along with the muscle as a free flap has been described earlier, reported for bridging calcaneal defect and small mandibular defects. The use of this flap as a source of free vascularised bone has not been widely practised since these initial few reports. Anatomical studies were carried out to assess the feasibility of using this flap for reconstructing maxillary and other head and neck defects, following which it was successfully used for these indications. The preliminary report describes the flap anatomy, method of harvest and its potential uses in head and neck reconstruction.

## INTRODUCTION

Tensor Fascia Lata (TFL) muscle has been well studied with regard to its anatomy and vascular supply, enabling its use as a pedicled muscle or musculocutaneous flap for reconstructing adjacent trunk defects. The muscle takes origin from the iliac crest, and this muscular attachment is the basis of its use as a composite flap incorporating vascularised bone. The iliac crest has been transferred as a pedicled vascular bone for treating vascular necrosis of the head of the femur.[1] Its use as a free bone-containing flap has been limited. Nahai[2] used it first for bridging a calcaneal defect successfully in two cases. Using it for the head and neck defects is reported only once in the literature. The bone was used in two cases for reconstructing mandibular defects, with one of the flaps failing to survive. Further reports on the use of this flap either for head and neck or elsewhere in the body are lacking. The present study was initiated to look at the possibility of using this flap with various combinations as a source of vascularised bone as well as other components for reconstruction of head and neck defects.

Cadaver dissection studies were performed to determine the morphological features and vascular anatomy. The flap was utilised successfully for the reconstruction of midface defects.

The purpose of this article is to provide a preliminary report discussing the flap anatomy, method of harvest and its potential uses in reconstructive surgery.

### Flap anatomy

TFL is a flat muscle in the upper part of the lateral thigh, taking origin from the outer lip of the iliac crest, lateral surface of the anterior superior iliac spine (ASIS), adjacent iliac bone and the deep surface of the fascia lata, and inserting into the fascia lata in the middle third of the thigh. It's artery arises from the ascending or transverse branch of the lateral circumflex artery, which is a branch of the profunda femoris artery, or rarely from the common femoral artery, entering the TFL muscle on its deep surface, close to the anterior border of the muscle, about 6–8 cm below the ASIS. The venous drainage follows the artery in the form of paired venae comitantes. The muscle is firmly fixed at its origin to the iliac crest and the outer table for a length of 4–5 cm and a vertical height of 3 cm; this bone being well nourished by the periosteal supply at this region of muscle attachment.

**Figure 1:**
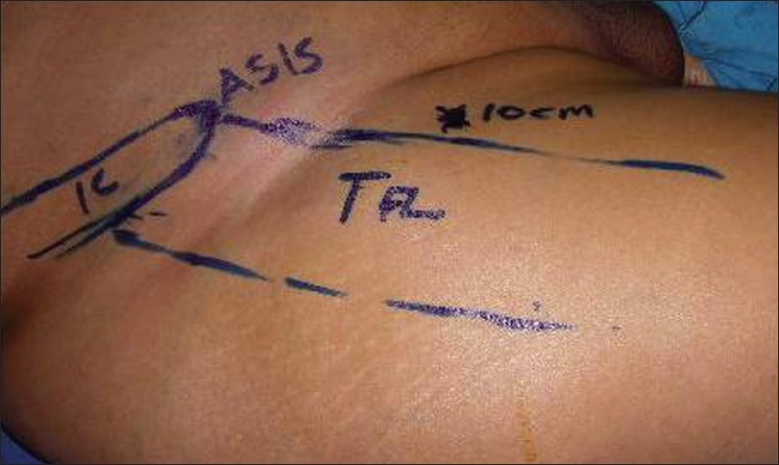
Muscle and its attachment to the iliac crest outlined in the patient. IC, Iliac Crest; TFL, Tensor Fascia Lata muscle; ASIS, Anterior Superior Iliac Spine

**Figure 2:**
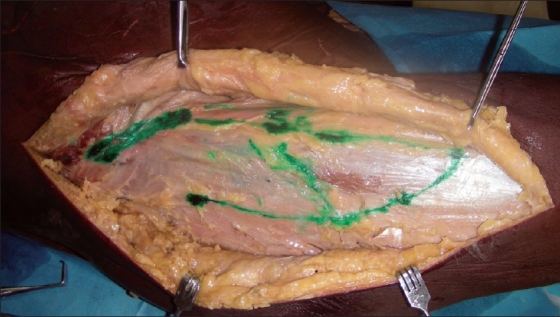
The flap outlined in the cadaver corresponding to the surface marking

Figure 1 shows the surface marking of the muscle and Figure 2 shows the corresponding details on the cadaver.

## OPERATIVE TECHNIQUE

The flap can be harvested in the supine position with a sand bag under the hip on the side of the flap, allowing harvesting of the flap and simultaneous head and neck resection by two teams.

**Figure 3:**
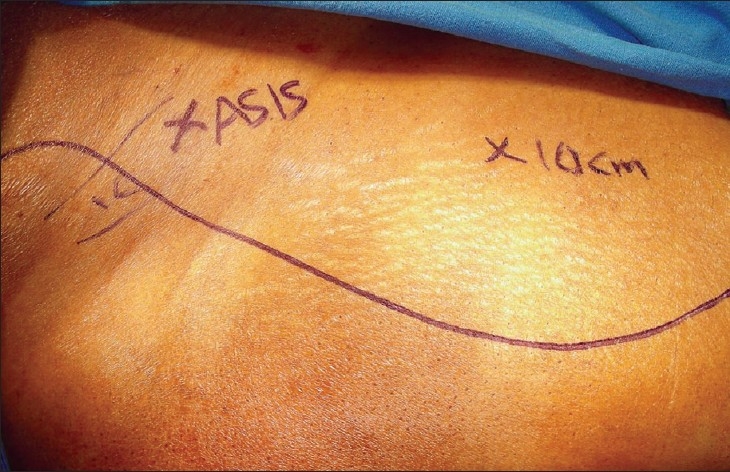
Incision marked when no skin paddle is needed

**Figure 4:**
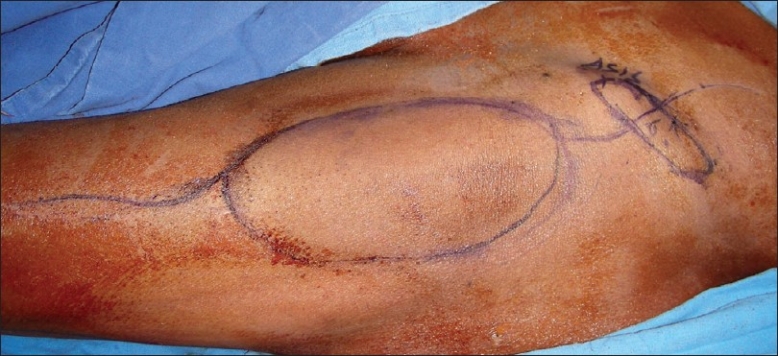
Flap with skin paddle marked

The skin incision depends on whether a skin paddle is needed or not. If no skin paddle is required, a lazy S incision is made from above the ASIS, passing in front of the ASIS and extending into the lateral thigh up to the midthigh level [Figure 3]. If a skin paddle is needed, it is outlined over the TFL muscle [Figure 4] and the incision is extended upwards and downwards for further exposure. The skin paddle can be sited anywhere over the TFL muscle or the adjacent fascia.

**Figure 5:**
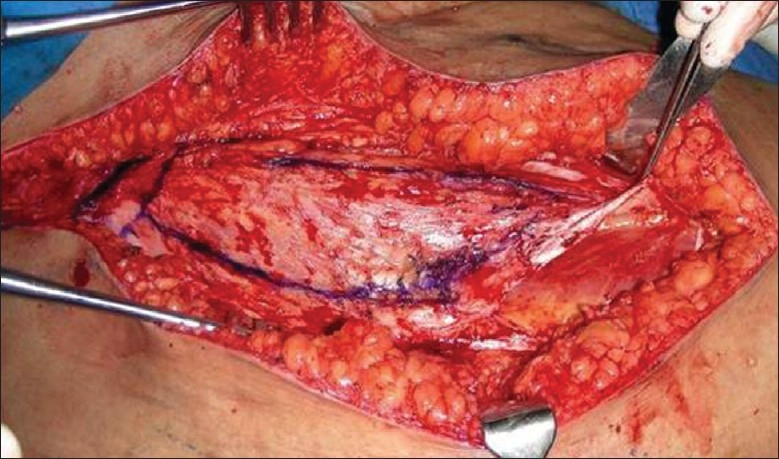
Tensor fascia lata muscle with its origin from the iliac crest exposed and outlined

**Figure 6:**
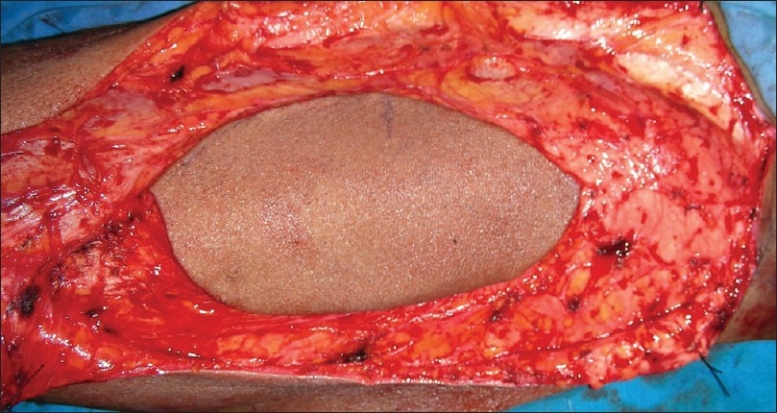
Skin flap and the surrounding muscle outlined

**Figure 7:**
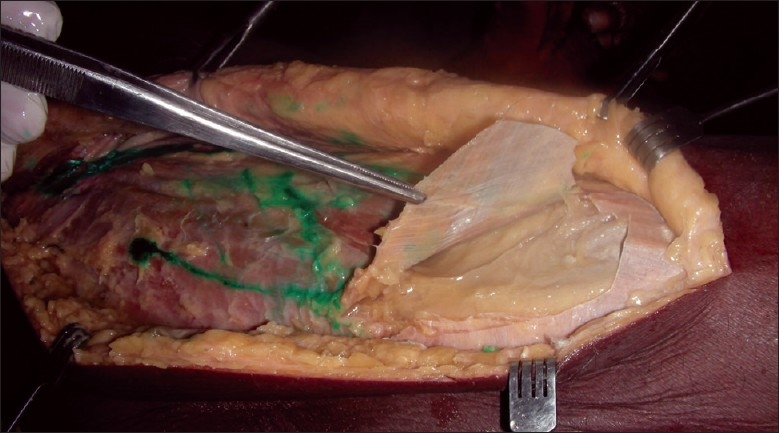
Dissection under the fascia lata shown in a cadaver

The skin flaps are raised all around so that the anterior third of the iliac crest, with its muscle insertions, the TFL muscle and a portion of the fascia lata are exposed [Figure 5]. If skin paddle is needed, the incision is made around it and the skin flap elevation proceeds in the usual way [Figure 6]. The fascia lata is now incised below the level of the muscle insertion and is lifted to get a plane under the muscle, with the dissection now proceeding upwards in this plane [Figure 7].

**Figure 8:**
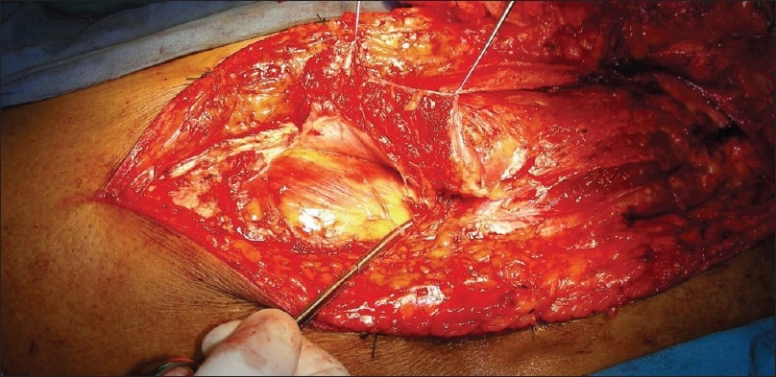
Muscle being lifted up

**Figure 9:**
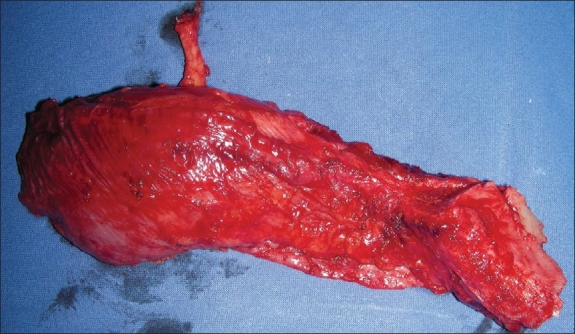
Harvested flap designed for orbitomaxillary reconstruction

The upward dissection proceeds along the anterior border of the muscle. The latter is dissected carefully to identify the vascular pedicle that passes over the vastus lateralis and enters the deep surface of the muscle. The vascular pedicle is identified entering the deep surface of the muscle 6–8 cm inferior to the ASIS. Attention is now brought to the dissection along the posterior surface of the muscle, which needs sharp dissection to free it from the underlying gluteus medius muscle. There is a constant branch from the pedicle that traverses laterally through the gluteus medius muscle at about 6 cm from the anterior edge of the iliac crest, which needs to be clipped. The muscle attachment to the iliac crest is delineated anteriorly and posteriorly. On the superior aspect, the attachments of the abdominal wall muscles are incised and the medial surface of the iliac crest is sufficiently bared. Cuts are made in the iliac crest to include the bone in the flap, with the anterior cut being placed just posterior to the attachment of the sartorius muscle. The horizontal cut in the deeper aspect is made at 4–5 cm depth incorporating the full thickness of the ilium. (An attempt was made to harvest only the outer table along with the crest in two cases. However, since this was found to be technically difficult, full thickness bone harvest is suggested.) The length of the bone harvested can be 6–7 cm, which corresponds to the area of muscle attachment and 2–3 cm beyond it. After the entire flap is dissected out, the pedicle is traced deep to the rectus femoris to its origin from the lateral circumflex vessels. The harvested flap is shown in Figures 8 and 9.

The pedicle length ranges from 6 to 8 cm. The pedicle length can be increased nominally by dissecting the lateral circumflex artery proximally. The calibre of the vessels matches well with that of the neck vessels. The bulk of the muscle inferior to the entry of the pedicle can be trimmed to allow the pedicle to be tunneled to the neck. Also, intramuscular dissection of the pedicle helps to reduce the bulkiness at the tunnelling area.

## RESULTS

**Figure 10:**
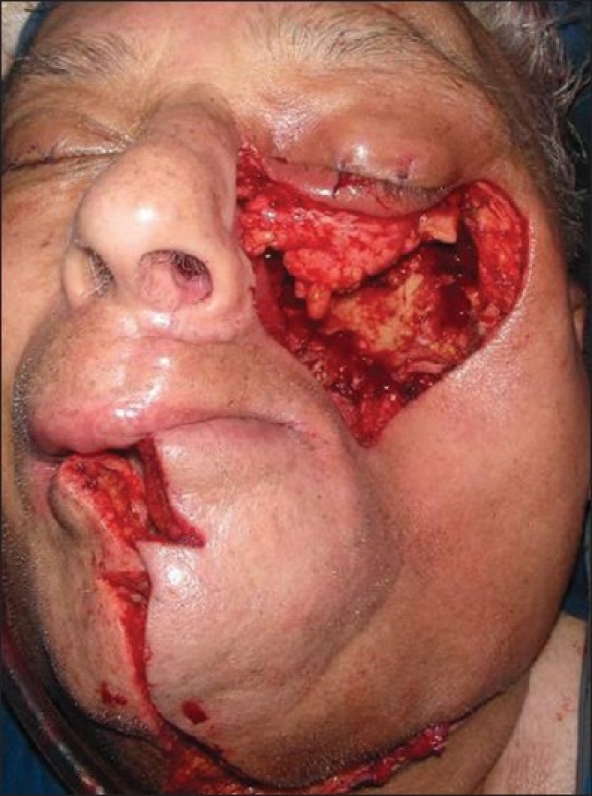
Intraoperative photograph of an orbitomaxillary defect

**Figure 11:**
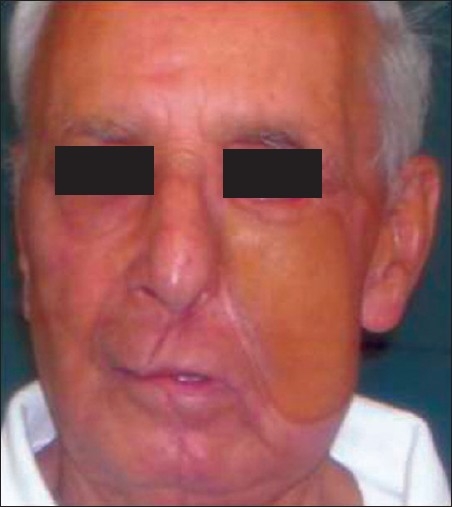
Postoperative photograph of the same patient showing a well-settled skin paddle

**Figure 12:**
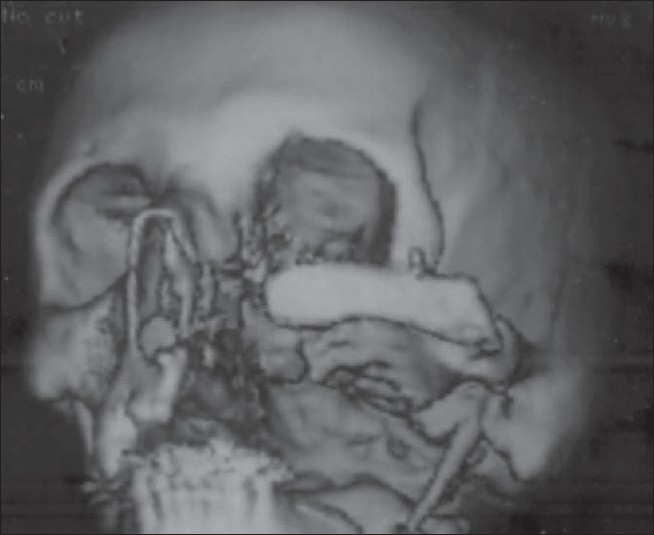
Computed tomography scan of the same patient 6 months postoperatively showing well-incorporated bone

**Figure 13:**
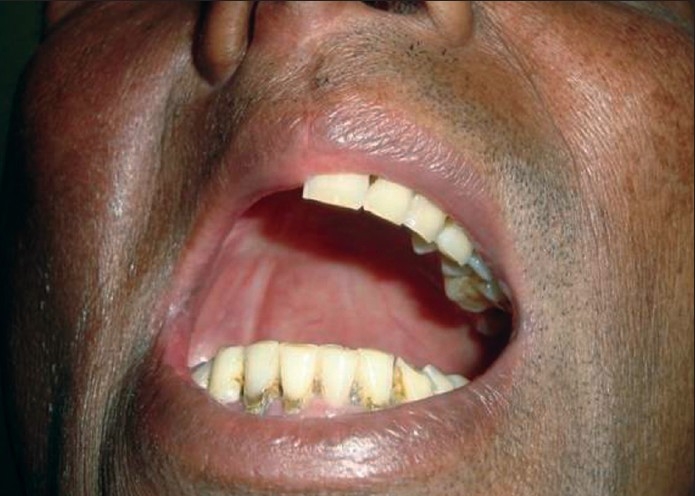
Intraoral view to show the healed palatal defect in another patient

Thus far, the flap has been used successfully in 10 cases for various head and neck defects, of which there was one failure due to anastomotic problems. In another case, there was partial necrosis of the bone. Figures 10‐13 show the 6-months postoperative result of two patients who had maxillary reconstruction using the flap, and the computed tomography scan. Predominantly, the flap has been utilised for orbitomaxillary reconstruction and detailed analysis of this series is under preparation for publication.

## DISCUSSION

TFL muscle or musculocutaneous flap has been regarded by clinicians as a versatile and reliable tool to cover pressure sores of the sacrum and ischium and defects of the abdominal wall and groin.[3‐8] Initially described as a pedicled flap, it has also been used as a free flap for covering defects elsewhere in the body. The attached portion of the iliac crest was used as a pedicled vascularised bone flap by Davies and Taylor[9] in an animal study followed by its successful use in clinical cases by Davies[10] and Chacha[1] to provide vascularity to the femoral neck. A free TFL iliac crest flap was used as free bone flap initially by Nahai[2], when he used it for reconstructing calcaneal bony defects. The only report of this flap being used as a free bone flap for head and neck reconstruction is by Baker[11] in 1992. He used it for reconstructing a segmental mandibular defect resulting from excision of cancer in two patients. The first flap survived whereas the second one failed during the immediate postoperative period.

The TFL muscle is usually classified as Type I, with one major vascular pedicle (Mathes and Nahai);[12] but, at least in 20% of the cases, the flap could be type II, with one dominant and another minor pedicle, as demonstrated by the cadaver studies of Saadeh.[13] The blood supply of the flap has been described as being through the lateral circumflex branch of the profunda femoris artery. The lateral circumflex artery gives off two or three branches, namely the ascending and the descending branch and, occasionally, the transverse branch. The frequency of the branches supplying the muscle, is reported by Saadeh in their elegant study on hundred cadavers. In 67% of the cases, the supply was by a single dominant branch from the ascending branch. In 13%, another branch arising from the same provided a second supply entering the middle third whereas in 20% of the cases, the second vessel arose from the descending branch and was destined to the lower third of the muscle.

There are a lot of advantages for this flap over the other composite vascular bone flaps. The flap anatomy is constant and the harvesting technique is easy to master. The components of the composite flap can be bone and muscle, bone, muscle and skin over muscle and skin only. The flap provides good-quality corticocancellous bone, although in a limited quantity. The donor site morbidity is minimal due to the preservation of the integrity of the abdominal wall muscles, making it superior to the traditional Deep Circumflex Iliac Artery (DCIA) based iliac crest flap. The shape of the iliac crest bone harvested fits the contours of orbital floor or the upper alveolus. The robust segment of muscle and skin allows simultaneous reconstruction of the palate as well as external skin defects.

**Figure 14:**
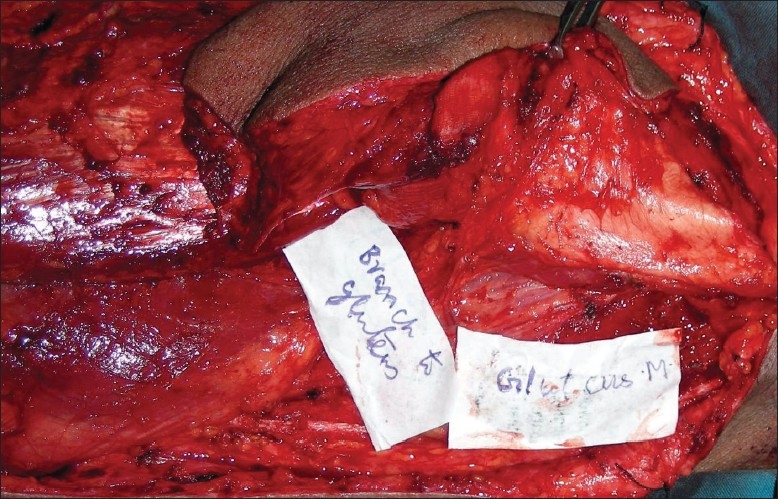
Dissection showing the constant branch to the gluteus medius

The other possible indications for the use of the flap would be in the lower limb defects with small bony gap and large soft tissue loss. The entire TFL muscle with or without the overlying skin is available to cover relatively large soft tissue defects. In the head and neck, whether mandibular reconstruction can be possible with this flap is a question worth pursuing, even though the initial report by Baker was disappointing. The flap may be suitable for reconstructing small defects of the mandible with external skin and soft tissue loss. Given the structure of the flap, it may be difficult to use it in presence of large mucosal defects. The vascular part of the bone may extend only for up to 5–6 cm and hence longer segmental defects may not be suitable for reconstruction by this flap. The constant branch to the gluteus medius [Figure 14] may be retained and dissected, allowing the harvest of a longer segment of bone. This will need confirmation by further dissection studies.

**Figure 15:**
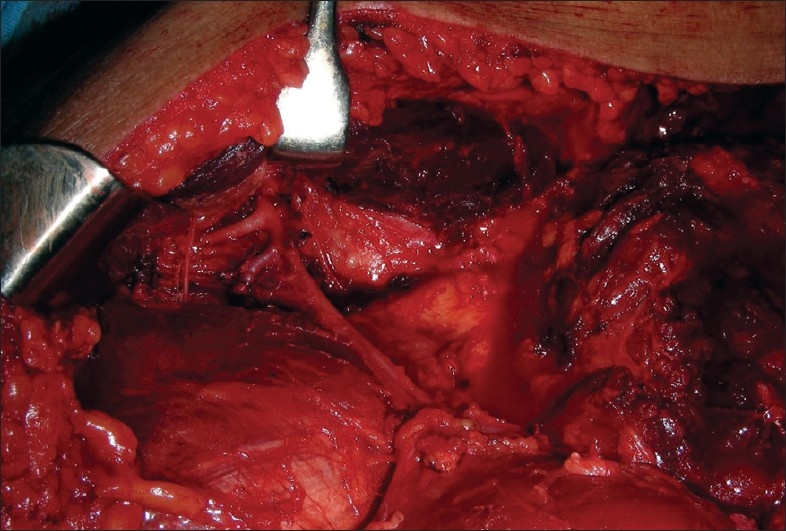
The branching pattern at the hilum of the muscle

The pedicle length varies from 6 to 8 cm and will allow direct anastamosis to the neck vessels. The muscle is bulky at the area of entrance of the vessels, which may create problems for passing the pedicle through the tunnel in the cheek when the flap is used for maxillary reconstruction. Intramuscular dissection of the pedicle and discarding the excess bulky muscle may be an answer. However, since the vessels divide into multiple branches before entering the muscle in many cases, as described by Saadeh [Figure 15], this may make this dissection difficult. This too needs to be confirmed by further dissection studies.

## CONCLUSION

TFL has been well established as a muscle and musculocutaneous free flap. Including the iliac crest as a vascularised bone component, even though it has been described previously, has not been found to be used extensively. This flap is a safe and robust method to transfer part of the attached iliac crest, especially to cover the maxillary and orbital defects. The flap anatomy makes it easy to raise and master the technique.

## References

[1] Chacha PB (1984). Vascularised pedicular bone grafts. Int Orthop.

[2] Nahai F, Hill L, Hester TR (1979). Experiences with the tensor fascia lata flap. Plast Reconstr Surg.

[3] Hill HL, Nahai F, Vasconez LO (1978). The tensor fascia lata myocutaneous free flap. Plast Reconstr Surg.

[4] Nahai F, Silverton JS, Hill HL, Vasconez LO (1978). The tensor fascia lata musculocutaneous flap. Ann Plast Surg.

[5] Nahai F (1980). The tensor fascia lata flap. Clin Plast Surg.

[6] McGregor JC, Buchan AC (1980). Our clinical experience with the tensor fasciae latae myocutaneous flap. Br J Plast Surg.

[7] Little JW, Lyons JR (1983). The gluteus medius-tensor fasciae latae flap. Plast Reconstr Surg.

[8] Luscher NJ, de Roche R, Krupp S, Kuhn W, Zach GA (1991). The sensory tensor fasciae latae flap: A 9-year follow-up. Ann Plast Surg.

[9] Davies JB, Taylor AN (1952). Muscle pedicle bone grafts: Experimental study. AMA Arch Surg.

[10] Davies JB (1954). The muscle-pedicle bone graft in hip fusion. J Bone Joint Surg Am.

[11] Baker SR (1981). Reconstruction of mandibular defects with the revascularized free tensor fascia lata osteomyocutaneous flap. Arch Otolaryngol.

[12] Mathes SJ, Nahai F (1981). Classification of the vascular anatomy of muscles: Experimental and clinical correlation. Plast Reconstr Surg.

[13] Saadeh FA, Haikal FA, Abdel-Hamid FA (1998). Blood supply of the tensor fasciae latae muscle. Clin Anat.

